# National and International Meat Inspection

**Published:** 1892-05

**Authors:** W. L. Williams


### NATIONAL AND INTERNATIONAL MEAT INSPECTION.

BY W. L. WILLIAMS.

*A Review of Prof. Schwartzkopff's Criticisms* (JOURNAL *Vol. XIII., p. 107*) *upon the Report of the Committee on Meat Inspection* (*Ibid., Vol. XII., p. 529*) *to the United States Veterinary Medical Association, Sept., 1891.*

The presentation of a paper on National and International Meat Inspection to the United States Veterinary Medical Association, at its annual meeting in Chicago in 1890, by Prof. O. Schwartzkopff, created a sufficient amount of interest to lead to the appointment of a special committee, consisting of Drs. Williams, Schwartzkopff and Clement, in order that the subject should again be brought before the association for further discussion.

Owing to a misunderstanding as to the *personnel* of the committee, Dr. Clement did not become aware of his connection therewith until too late to take an active part; and there being some differences of opinion upon essential questions between Dr. Schwartzkopff and the chairman, it was found expedient to make a report as chairman of the committee, at the same time requesting Prof. S. to prepare a special report upon the points in controversy.

When the committee came to report Prof. S. was unexpectedly absent, and this, with a want of time for proper discussion, prevented its consideration beyond the presentation of the report, and again the recognized importance of the questions involved led to the appointment of Drs. Schwartzkopff, Faville and Bryden as a special committee for 1892.

For reasons at present unknown, Dr. Schwartzkopff has seen fit, instead of awaiting his opportunity at the next meeting of our association, to enter upon an exhaustive criticism (JOURNAL, Vol. XIII., p. 171) of the report (Ibid., Vol. XII., p. 529) at every point where it at all diverged, either in form or spirit, from his paper presented in 1890. (Ibid., Vol. XI., p. 599.)

In attempting to reply, it is desired that the brief quotations made by Dr. S., from the report, be read in their original text, with their connections, since in one or two instances false conclusions may readily be drawn from such extracts.

In criticising the suggested classification of diseased meats (Ibid., Vol. XII., p. 534), and attempting to uphold that proposed by him (Ibid , Vol. XI., p. 601), he states that such a classification as that of the report was proposed long ago by "dreamers" but failed. He neglects to state where and when it was tried, or why it failed. Neither does he apparently consider that even if we now possess sufficient scientific data upon which to base such a classification, we did not at the time to which he refers. It certainly cannot well be held that because a premature attempt at scientific classification, probably tried under strong prejudice and unfavorable surroundings failed, that a scientific classification of diseased meats is still impossible. The impossible of yesterday need not be the impossible of to-day. Hence it was urged in the report that something more scientific should be possible now than that which has long been in use.

In stating that the proposed classification did not rest upon facts, and was, therefore, not scientific, Dr. S. denies the truth of four distinct propositions which we had been led to believe were universally admitted by scientific veterinarians. They are :

1st. Meat infested by certain living animal parasites, when eaten by man is dangerous to health or life through the development or migration, or both, of the parasitic organism.

2d. There exist diseases of animals not transmissible to man, which can only injure the consumer of the flesh through the presence of chemical products, either the result of special micro-organisms or of tissue metamorphosis.

3d. There are animal diseases transmissible to man directly, and bearing in the flesh a second element of danger to the consumer in the presence of ptomaines or other topic products.

4th. There exist bacteriological diseases of animals identical with certain diseases of man, which, in individual cases, may affect the animal in so mild a form as to be dangerous to man only from the presence in the flesh consumed of living micro-organisms capable of transplantation.

If these propositions are facts, a classification of diseased meats upon these is scientific, but if Dr. S. can show that there are errors, then the proposed classification cannot ensue.

Supplementary to his charge that the proposed classification is unscientific, he misconceives the force of the word *may* as used in a piecemeal quotation from the report, and gives it a meaning not at all intended, and which we believe cannot be substantiated by a strict English construction of the sentences.

In attempting to justify his classification in the paper presented in 1890 (JOURNAL, Vol. XI., p. 601), he presents as his sole argument that it is the method generally employed in Germany, "and has grown up from established facts and in conformity with practical principles." He fails to note that the environments in Germany are quite different from those of meat inspection in America, so that established facts, etc., may be very widely different, and what may be necessary or advisable in Germany does not mark out an essential line for action in America.

Preliminary to the exceptions Dr. S. offers to the position taken in the report, relating to the transmissibility of actinomycosis, he objects to the definition of contagious (JOURNAL, Vol. XIII., p. 538) as "unnatural and strained (Ibid., Vol. XIII., p. 109), and denies that our conception of the word *contagious* has changed during the past two hundred years, but that it means now, as then, that "a healthy animal must come in contact with a diseased animal to contract a particular disease."

To say that our conception of contagious has been stationary for two centuries is practically equivalent to saying that medical science has been sleeping during that time, for if we have made any advances at all, if we have discarded older conceptions and applied newer and more scientific meaning to words, surely our conception of contagious (or infectious) has undergone a wonderful evolution. But contagious does not bear to us the same conception as two centuries ago, and did not mean then, that in order for an animal to contract a contagious disease it must come in contact, or even near to, one affected with the same malady, and even writers of the present generation have not so held. Fleming maintained in his *Veterinary Sanitary Science and Police* that glanders could originate spontaneously, *i. e.*, other benign diseases, like strangles, etc., could, under certain conditions, develop into glanders ; but Dr. Fleming now admits that medical science grows, and cheerfully owns that he erred and that his conception of contagion is far different now from what it was two or three decades ago ; and his admission represents, not the views of a misguided individual, but an enormous change in the knowledge of the medical world and a total reconstruction of our conception of contagion.

Even now no authority holds that contact of animal with animal, as stated by Dr. S., is at all necessary for transmission of contagious diseases, as the diseased animal may be left far behind and the disease be transported across continent and ocean and planted anew in the bodies of susceptible animals.

Nor do we now mean that the disease-producing material must be derived from a diseased animal, but can procure it from the bacteriological laboratory, where it has been cultivated, generation after generation, on vegetable or other media, or from the soil in certain localities, where, so far as is now known, the pathogenic germs have existed ages before the animals, now regarded as susceptible, had appeared in the infected area.

In what respect the committee's definition of contagious was unnatural or strained he does not say, and leaves his bare assertion as his only argument.

Passing to what Dr. S. apparently considers gross ignorance on the part of the English-speaking veterinary profession in their general want of discrimination in the use of the terms *contagious* and *infectious*, he suggests that "there are surely yet, as before, contagious and infectious diseases, and the best pathologists still apply both terms in a well-defined way."

It must be frankly admitted that no English veterinarian has yet appeared who is able to draw a distinction between the two in a sufficiently clear manner to meet the approbation of a majority of our profession.

Turning to German veterinary literature, we find Professor Dieckerhoff and Professors Friedberger and Fröhner in their standard works on special pathology disregarding entirely the division into classes of transmissible diseases, the two latter explicitly stating that classification was useless, and placing the entire list of transmissible maladies described in index and text in one category, infectious krankheiten ; while the French writers apparently favor the word contagious, and I use it largely to indicate the same group of diseases.

We are well aware that certain writers have ineffectually (except for themselves) attempted to make a distinction between the two words by applying *contagious* to those affections requiring direct contact, and *infectious* to those in which no such contact is essential, while others reverse this order of application.

Various other pathologists hold yet other views as to the meaning of these words, and among these there is, according to Dr. S., a group, to which he apparently belongs, who apply the term *infectious* to those diseases in which "the micro-organism has its natural origin outside the animal body on vegetable life" By such a classification apparently, Dr. S. holds that actinomycosis, for instance, is not contagious, but merely infectious, and that the actinomyces originate outside the animal body on vegetable life. The proof of such genealogy for actinomyces apparently rests upon the fact that the germ was studied for years as affecting the animal body before it was found in vegetation, and it still exists in a more luxuriant form in the animal than on plants. Being by the admission of Dr. S. transmissible from animal to animal by inoculation, the disease was, according to the above views, *contagious* when known in animals only, and became *infectious* when found some years later growing very scantily on vegetation. But a large proportion of known pathogenic germs are artificially cultivated in vegetable media, and we at present have no means of knowing but that they were originally derived from vetegtation, consequently such a classification would prove vacillating, and the line separating the two would weaver two and fro as the weight of evidence at a given epoch favored an original animal or vegetable habitat, and could never be definitely settled until new means are discovered for learning the original source and habitat of living organisms of every form and kind. Priority of source of discovery by man, or complexity of development, cannot serve to positively indicate the primitive habitat. Consequently, we see utter confusion in the various attempts to attach to these words a satisfactory and definite meaning.

If, however, one will at the outset define the terms to be used, his definition must necessarily suffice for his contribution, and no difference how utterly unlike may be the meaning given the word by others, critics are in duty bound to base their criticisms upon the word as defined by the writer, and not upon what it means to them.

With this end in view, the committee defined early the word *contagious* so that all might know, not what this or that man meant, but what the committee meant by that word, thus confining the meaning of the word in subsequent portions of the report to the signification expressly given it in the definition.

If Dr. S. has effectually demolished the definition of *contagious*, his criticisms of subsequent portions of the report are wholly irrevelant as the report ends in so far as the word *contagious* is involved when that definition is destroyed, while if the definition survive the criticism must be based thereon.

There is manifestly nothing to be gained by Dr. S. accusing the committee of misstating the views of numerous authors on the subject of actinomyçosis (JOURNAL, Vol. XIII., p. 175), when the sole ground for such accusation rests upon the different conceptions held by the disputants as to the meaning of a word the force and meaning of which, as used in the report, had already been clearly stated. The real foundation almost throughout Dr. Schwartzkopff's criticisms rests upon this quibble over the meaning of a word.

Dr. S. criticises the committee sharply for concluding that inoculation constitutes a crucial test of contagiousness, and asserts that pleuro-pneumonia of cattle "has never been transmitted to any experimental animal." The point seems, however, to be in controversy, and Arlving (*Vet. Jour.*, Fleming's, Vol. XXIX., p. 260) and Law (*Farmer's Vet. Adviser*, Appendix, p. 78) seem to hold the opposite view. Fleming (*Vet. San. Sci. and Police*) seems strongly inclined also to believe the disease transmissible by inoculation.

Moreover, Dr. S. will doubtless admit that inoculation produces a distinct degree of immunity against this disease, and in the present state of our knowledge this immunity can be referred to but two sources—the growth within the body of the inoculated animal of the pathogenic germ, or to the introduction into the healthy animal of toxic products resulting from bacterial growth or presence. Admit the former, and the immunized animal had pleuro-pneumonia; admit the latter, and it was not inoculated with the disease-producing germ. The above reasons proven incompetent, the committee's definition of *contagious* would still defeat the argument of Dr. S., and properly hold that the test of contagiousness is not made complete until the inoculation is pulmonary instead of caudal.

On the other hand, to show that all inoculable diseases are not contagious, he cites the pus-forming germs, but a casual glance of the definitions given by both Dr. S. (Journal, Vol. III., page 109), and the committee (Journal, Vol. XII., p. 538) will at once show that this fulfils all the conditions of either, and furthermore, all modern surgery is based upon the transmissibility from patient to patient of this pathogenic micro-organism.

While not definitely stated, the inference may be fairly drawn from the criticisms of Dr. S. that he would have the reader believe that the committee held in their report that actinomycosis was highly contagious; that it was generally due in animals to intertransmission; that it could be readily transmitted from animal to animal, or animal to man, and that the destruction of the meat from animals mildly affected was an advisable sanitary measure.

A careful reading of the full text of committee report will clearly show these inferences to be wholly without foundation in fact.

